# Small Split Doses of CD34^+^ Peripheral Blood Stem Cells to Support Repeated Cycles of Nonmyeloablative Chemotherapy

**DOI:** 10.1155/2017/4184879

**Published:** 2017-11-12

**Authors:** Maxim Yankelevich, Sureyya Savasan, Igor Dolgopolov, Roland Chu, George Mentkevich

**Affiliations:** ^1^Division of Hematology/Oncology, Department of Pediatrics, Children's Hospital of Michigan, Wayne State University, 3901 Beaubien Street, Detroit, MI 48201, USA; ^2^Institute for Pediatric Oncology, N.N. Blokhin Russian Cancer Research Center, 24 Kashirskoye Shosse, Moscow 115478, Russia

## Abstract

Cumulative myelosuppression is the main limiting factor for administration of repeated cycles of chemotherapy. We present a case series of five pediatric patients with high-risk solid malignancies who received small split peripheral blood stem cells (PBSC) doses of less than 1 × 10^6^/kg CD34^+^ cells obtained after a single leukapheresis procedure and given after repeated cycles of ICE (ifosfamide, carboplatin, and etoposide) chemotherapy. Mean duration to absolute neutrophil count (ANC) recovery to >1000/mm^3^ and platelet recovery to >50 × 10^3^/mm^3^ was 17.1 and 24.3 days. Using split doses of PBSC prevented prolonged neutropenia after repeated cycles of submyeloablative chemotherapy.

## 1. Introduction

Cumulative myelosuppression is the main limiting factor for administration of repeated cycles of intensive chemotherapy such as ICE when patients can tolerate only a limited number of full-dose chemotherapy cycles even with the use of growth factors [1].

One strategy to reduce hematological toxicity is the infusion of autologous PBSC after chemotherapy. The minimal recommended dose of hematopoietic progenitors for successful transplantation after myeloablative chemotherapy was described as 2 to 2.5 × 10^6^/kg of CD34^+^ cells [2].

Less is known about the minimal doses of CD34^+^ cells that would still support enhanced hematopoietic reconstitution after nonmyeloablative conventional therapy regimens. PBSC support at doses exceeding 2 × 10^6^/kg allowed maintaining dose intensity of nonmyeloablative conventional chemotherapy in patients with solid tumors [3–12]. Patients required multiple leukapheresis procedures to provide more than 2 × 10^6^/kg of CD34^+^ cells per cycle to support several chemotherapy cycles. Here, we report a retrospective analysis of our clinical experience where we used split doses of less than 1 × 10^6^/kg CD34^+^ cells obtained after one leukapheresis procedure to support repeated cycles of chemotherapy in pediatric patients with solid tumors.

## 2. The Case Series

Five patients were treated at the N.N. Blokhin Russian Cancer Research Center (Moscow, Russia) and the Children's Hospital of Michigan (Detroit, MI). We obtained informed consents for chemotherapy and PBSC collection from all patients included in this analysis. The patients received repeated cycles of ICE (ifosfamide 9000 mg/m^2^, carboplatin 500 mg/m^2^, and etoposide 500 mg/m^2^) chemotherapy supported by autologous PBSC. Three patients had a single leukapheresis procedure following 2nd to 4th cycle of chemotherapy and G-CSF stimulation, and the product was split into four equal doses. In two patients (patients 2 and 4, Table 1), we used PBSCs obtained after leukapheresis procedures that did not collect required numbers of CD34^+^ cells to support myeloablative chemotherapy and otherwise would be discarded. The PBSCs were reinfused 24 hours after completion of consolidation chemotherapy cycles followed by G-CSF or GM-CSF stimulation. We used the number of days from the start of chemotherapy to ANC recovery to > 1000/mm^3^ to evaluate hematopoietic toxicity. We compared these data to induction chemotherapy cycles administered without PBSC support in the same patients. We used the Student's *t*-test to determine the significance of differences.

The following is a short description of each patient's case:

Patient 1 was a 2-year-old male with stage 4 anaplastic Wilms' tumor whose metastatic lung disease was refractory to the first line of chemotherapy, and he was switched to ICE. He subsequently received 4 cycles of ICE chemotherapy with PBSC support after cycles 3 and 4. He had partial radiological response (PR) to chemotherapy and subsequently received high-dose melphalan with PBSC support; however, he developed a second disease recurrence 3 months after transplant.

Patient 2 was a 17-year-old female with stage 4 favorable histology Wilms' tumor who developed both pulmonary and abdominal recurrence 10 months after initial therapy. She was treated with abdominal tumor resection and 4 cycles of ICE chemotherapy with PBSC support after cycle 3 (an infusion of 0.3 × 10^6^ CD34^+^ cells/kg from apheresis that did not collect required dose for myeloablative therapy was given). She had PR to chemotherapy. She then received high-dose thiotepa and melphalan with autologous bone marrow transplant. She developed recurrent disease 4 months after transplant.

Patient 3 was a 9-year-old male with stage 3 favorable histology Wilms' tumor who had pulmonary recurrence 13 months after his initial therapy and was treated with 6 cycles of ICE chemotherapy with PBSC support after cycles 3 through 6. He had PR to chemotherapy and was alive without signs of disease more than 10 years off therapy at the time of this report.

Patient 4 was a 1.5-year-old male with stage 3 Wilms' tumor abdominal recurrence. He underwent complete abdominal tumor resection and then received ICE chemotherapy as a consolidation with PBSC support after cycle 3 (an infusion of 1.8 × 10^6^ CD34^+^ cells/kg from apheresis that did not collect required dose for myeloablative therapy was given). The patient was still under therapy at the time of this report.

Patient 5 was a 20-year-old female with metastatic PNET with primary abdominal tumor and multiple metastases to the lungs, lymph nodes, and vagina. She underwent abdominal surgeries and received ICE chemotherapy. She had very good partial response after the first two cycles but developed significant hematological toxicities after cycles 3 and 4 even with 30% chemotherapy dose reductions. She received her ICE cycles 5 and 6 at full dose followed by 0.82 × 10^6^/kg CD34^+^ cells support per cycle and tolerated them well with significant reduction of hematological toxicity. The ANC recovery to > 1000/mm^3^ was on day 26 after cycle 4 and on day 17 after cycles 5 and 6. Platelet count recovery to > 50,000/mm^3^ was on day 29 after cycle 4 and on day 26 after cycles 5 and 6. She subsequently remained in remission for 7 months but developed metastatic recurrence thereafter and died of disease.

Severe myelosuppression was the main toxicity observed in all patients receiving ICE chemotherapy. The doses of infused CD34^+^ cells ranged 0.3 to 1.8 × 10^6^/kg (mean 0.76 × 10^6^/kg), and in 9 out of 10 PBSC infusions, the dose of CD34^+^ cells was below 1 × 10^6^/kg (Table 1). In patients who started to receive PBSC support after their 3rd and subsequent cycles, there were no significant differences in ANC recovery between the first 2 induction cycles and the subsequent cycles given with PBSC support (17.6 days after cycles 1 and 2 versus 17.1 days after cycle 3 and subsequent cycles, *p*=0.28). All PBSC-supported cycles were given at the full planned doses.

## 3. Discussion

ICE chemotherapy is one of the effective regimens in pediatric solid tumors [13–16]. However, dose intensity of repeated cycles decreases due to hematological toxicity: multiple publications report the necessity to de-escalate chemotherapy dosing after the third and subsequent cycles of ICE regimen [1, 13, 15, 16], and while median days to ANC and platelet recovery after the first 2 cycles were reported around 18 and 22 days, respectively [1, 13, 14], recovery times significantly increase after subsequent cycles. Thus, Yankelevich et al. [14] reported 26 and 30 median days to ANC and platelet recovery following the second two cycles of ICE. In this report, we demonstrate that split PBSC doses obtained from one leukapheresis procedure and containing less than 1 × 10^6^ CD34^+^ cells per kg provide sufficient support for ANC recovery after repeat cycles of ICE chemotherapy. Several reports described PBSC support at a traditional dose range of >2.5 × 10^6^/kg. Hawkins et al. [6] and Bensimhon et al. [8] used at least 2–2.5 × 10^6^/kg CD34^+^ cells per cycle after several cycles of intensive chemotherapy similar to ICE in children with solid tumors. To collect these cell numbers, patients had to have multiple (up to 6) leukapheresis procedures. The average number of days to ANC recovery to > 500/mm^3^ was 15–17 [6]. Bensimhon et al. showed that after giving 4.9 to 10 × 10^6^/kg of stem cells after 3rd cycle of cyclophosphamide/carboplatin, the median time to ANC recovery to > 750/mm^3^ ranged from 14 to 16 days [8]. These parameters of hematological recovery are similar to our data obtained with much smaller doses of CD34^+^ cells (Table 1).

Leukapheresis is an expensive procedure commonly requiring central line placement and additional use of growth factors to mobilize stem cells. It takes multiple leukapheresis procedures over several days to collect the desired CD34^+^ cell dose [6–8, 10]. Using small split doses of PBSC may provide effective support for multiple cycles of conventional chemotherapy. CD34^+^ stem cell doses below 2 × 10^6^/kg are safe and may be efficient after conventional cycles of chemotherapy providing protection from cumulative myelotoxicity.

## Figures and Tables

**Table 1:** PBSC support and hematopoietic recovery after postinduction cycles of ICE; comparison of induction and postinduction cycles.

Pts. no.	Cycle no.	Chemotherapy	Number of CD34^+^ cells × 10^6^/kg	Days to ANC > 1000/mm^3^	Days to Plt > 50 × 10^9^/L	Fever
1	3	ICE + GM-CSF	0.35	18	20	ND
	4	ICE + GM-CSF	0.35	23	34	Yes
2	3	ICE	0.3	17	21	No
3	3	ICE + G-CSF	0.8	16	22	No
	4	ICE + G-CSF	0.8	16	22	No
	5	ICE + G-CSF	0.8	16	25	No
	6	ICE + G-CSF	0.8	16	25	Yes
4	3	ICE + GM-CSF	1.8	15	22	ND
5	5	ICE + G-CSF	0.82	17	26	No
	6	ICE + G-CSF	0.82	17	26	Yes
Mean	—	—	0.76	17.1	24.3	—

Hematopoietic toxicity		ICE cycles 1 and 2 (*n* = 10)	ICE cycle 3 and subsequent cycles with PBSC support (*n* = 10)		*p*	

Mean days to ANC > 1000/mm^3^		17.6	17.1		0.28	

Mean days to Plt > 50 × 10^9^/L		21.8	24.3		0.053	

ND, no data available.
